# First records of *Ganoderma suae* and *Pleurotus tuber regium* in Vietnam

**DOI:** 10.1038/s41598-026-45075-2

**Published:** 2026-03-28

**Authors:** Luane Pieau, Tran Bao Tram, Xuan Binh Minh Phan, Khuc Thi An, Do Quynh Chi, Jean Baptiste Simurabiye, Le Thi Hoang Yen

**Affiliations:** 1 Université Lille, Boulevard Paul Langevin, Cité Scientifique, 59655 Villeneuve-d’Ascq, France; 2 Center for Experimental Biology, NACENTECH, MST, C6 Thanh Xuan Bac, Thanh Xuan, Hanoi, 10000 Vietnam; 3 Nha Trang University, Nha Trang, Khanh Hoa 10000 Vietnam; 4 High School for Gifted Students, Ha Noi Univertity of Science, Hanoi, 10000 Vietnam; 5 Hanoi University of Science and Technology (HUST), Hanoi, 10000 Vietnam

**Keywords:** Mushroom, Diversity, Newly recorded, Vietnam, Taxonomy, Biotechnology, Evolution, Microbiology, Plant sciences, Ecology

## Abstract

Two medicinal mushroom species, *Pleurotus tuber-regium* (*Lentinus tuber-regium)* (commonly known as the sclerotium-forming oyster mushroom) and *Ganoderma suae* (a resinous polypore recently described from Southeast China in 2024)—are reported for the first time in Vietnam. The taxonomic histories of both genera, *Ganoderma* and *Pleurotus* are complex due to morphologically similarities among closely related species, posing challenges for accurate identification. In this study, specimens collected from National resources of Vietnam were identified combining detailed morphological examination with molecular analysis using rDNA ITS markers for phylogenetic confirmation. This report enhances the understanding of fungal diversity in Vietnam and provides a basis for further studies on cultivation, bioactivity, and value-added applications.

## Introduction

*Ganoderma* is a widespread genus of basidiomycetous fungi characterized by tough, leathery to woody basidiocarps known as polypores. Species within this genus exhibit diverse ecological roles, growing as biotrophs on living trees or necrotrophs on dead trees, logs, and in swampy environments. They are often plant parasites, contributing to white rot, root and stem rot, and wood decay, which can severely compromise host plant health. They live in hot and humid environments and so can easily grow in tropical and subtropical regions^1–3^.

The genus was first established in 1881 by Petter Adolf Karsten for the laccate, stipitate, white-rot fungus *Polyporus lucidus*. Subsequently, the taxonomic circumscription of the genus has undergone substantial changes as more species with double-walled basidiospores were discovered. Nevertheless, numerous laccate and non-laccate species show overlapping morphological traits, and historical species descriptions were often incomplete, leading to decades of taxonomic ambiguity^2^.

To overcome these limitations, recent studies have successfully combined morphological observations with multilocus phylogenetic analyses—including ITS, nLSU, rpb2, tef1, mtSSU and nSSU—to redefine taxonomic boundaries within Ganodermataceae and to recognise new species within *Ganoderma*. A total 146 species in Ganodermataceae with available DNA sequences were involved in the phylogenetic analyses. Among them, 14 genera and 278 species were confirmed within Ganodermataceae^3,4^. Continuing progress in this direction has led to the description of additional taxa, recent estimates suggest the genus comprises 191–400 species of ecological and economic importance worldwide^3–5^.

*Ganoderma suae* was recently described from Southeast China by He et al*.*^6^ as a distinct lineage within the *G. resinaceum* complex based on morphological evidence and molecular phylogeny. The specific epithet “suae” honors the Chinese mycologist Su Ai-Ping, reflecting its discovery in subtropical forest habitats. Morphologically, *G. suae* resembles *G. resinaceum* and related taxa by its reddish-brown, varnished basidiomata and resinous context, but differs in pore size, spore dimensions, and the phylogenetic placement based on multigene analyses (ITS, LSU, and TEF1-α)^6^. The recognition of *G. suae* highlights the ongoing taxonomic complexity within the *G. resinaceum* group, where morphological convergence and incomplete molecular data have long obscured species boundaries^3^.

Vietnam, as a tropical country with diverse forest ecosystems, possesses substantial potential for fungal diversity, particularly within the genus *Ganoderma*. To date, fourteen species of *Ganoderma* have been documented from Vietnam^7–13^. Despite this remarkable diversity and the long-standing traditional use of *Ganoderma* species as medicinal mushrooms in Vietnam, *Ganoderma suae* has not previously been documented in the country. The present study therefore represents the first confirmed report of *G. suae* from Vietnam, thereby expanding the known geographical distribution of the species and contributing additional morphological and molecular data to support future taxonomic and biodiversity studies on *Ganoderma.*

*Pleurotus tuber-regium* (Fr.) Singer is a basidiomycete fungus with a complex taxonomic history, having been placed in several different genera due to its morphological characteristics, which share features typical of “oyster mushrooms” (*Pleurotus*) as well as cap–stipe structure and hyphal systems resembling those of the genus *Lentinus*. The species was originally described as *Agaricus tuber-regium* Fr. in 1821 and later transferred *Lentinus* ssp. (indexfungorum)^14^. In the following decades, it was transferred to *Pleurotus* by Singer (1949) and Neda and Nakai^15^ based on the morphology of the fruiting body, gill structure and molecular studies. This species is distinguished by the production of a large sclerotium, which may reach up to 30 cm in diameter—an uncommon trait within the genus *Pleurotu*s^15^. The sclerotium not only enables survival under harsh environmental conditions but is also widely used as food and traditional medicine in parts of Africa and Asia, where it has been associated with antioxidant activity, immune enhancement, and gut health benefits^16–19^.

## Material and methods

### Isolation and morphological identification

#### Sample and culture isolation

Sampling was carried out in Cao Bang and Nha Trang, Khanh Hoa of Vietnam. Nui Thung, Cao Bang is composed of a temperate rainforest, with over 200 plant species, including a mixed forest canopy consisting of broadleaf evergreen and tropical needleleaf trees. The sampling location was (22.78182^o^ N, 106.32046^o^ E), at a sampling height of 1000 m. The temperature was 27.5–28 °C, humidity was 85–84.2%. Time for sampling was in May 2023.

In Nha Trang, Khanh Hoa mushroom collection took place at (12.30092^o^ N 109.21533^o^ E) and (18.96472^o^ N, 109.80316^o^ E),150 m above sea level. Temperature was 27.0–29.6 °C. Humidity was 81.5%. Time of isolation: July 2022 and Oct. 2024.

To isolate the fungi, the fresh basidiomata were directly isolated from the fruiting bodies using the tissue method in the forest using potato dextrose agar (PDA) medium (Merck, Darmstadt, Germany). The plates were then transported to the laboratory and incubated at 25 °C for 7–10 days for culture isolation. The cultured strains were preserved in slant agar at − 80 °C in a sterilized glycerol-trehalose solution 10% glycerol and 5% trehalose.

#### Morphological study

Macromorphological characteristics of fresh material were observed. Specimens were described and photographed while fresh during daylight hours and observed under a Zeiss Stemi DV4 stereo light microscope (Carl Zeiss, Jena, Germany). Microscopic characteristics were based on dried specimens, and the freehand sections were mounted in 3% (w/v) KOH solution after staining with 1% (w/v) cotton blue in Melzer’s reagent. The measurements and line drawings of microscopic elements were made using the A2-Image program (Carl Zeiss). Basidiospore measurements were made from material mounted in Melzer’s reagent.

#### DNA extraction, polymerase chain reaction amplification, and sequencing

DNA was extracted from mycelia grown on PDA medium at 25 °C for 7 days using PrepMan Ultra Sample Preparation Reagent (Applied Biosystems, Foster City, CA). Polymerase chain reaction (PCR) was performed using a KOD-Plus Kit (Toyobo, Osaka, Japan) following the manufacturer’s protocol. The ribosomal DNA (rDNA) internal transcribed spacer (ITS) region was amplified with the primer pair ITS1 and ITS4^20^. Amplification of the DNA fragments was performed using the GeneAmp PCR System 9700 (Applied Biosystems). PCR products were checked by agarose gel electrophoresis and purified using an AMPureKit (Agencourt Biosciences, Beverly, MA). Sequencing reactions were performed by using the Big Dye Terminator version 3.1 Cycle Sequencing Kit (Applied Biosystems) and the same PCR primers.

#### Phylogenetic analysis

All of the sequences were assembled and edited manually using BioEdit version 7.09 (Tom Hall, Ibis Biosciences, Carlsbad, CA). The sequences were aligned with GenBank sequences retrieved from BLAST searches in the National Center for Biotechnology Information database (http://www.ncbi.nlm.nih.gov/) by using Clustal X^21^. All the sequences were assembled and edited manually using BioEdit V.7.09 (Tom Hall, Ibis Biosciences, Carlsbad, CA, USA).

Phylogenetic relationships of *Ganoderma suae* were reconstructed using Maximum Parsimony (MP) and Bayesian Inference (BI) based on the ITS dataset, following the analytical framework described by Fryssouli et al.^22^. The final alignment comprised 36 sequences and 559 characters, with Amauroderma rugosum (KJ531664; Cui 9011) designated as the outgroup (Table 1), consistent with previous phylogenetic treatments of Ganodermataceae^22^. For MP analysis, evolutionary rate heterogeneity among sites was modeled using a discrete gamma distribution with five categories (+ G; parameter = 3.1916) and a proportion of invariant sites (+ I; 66.16%), following standard phylogenetic procedures^23^. Bayesian inference was conducted in MrBayes v3.2.7^24^ under the GTR + Γ + I substitution model^25^. Two independent Markov Chain Monte Carlo (MCMC) runs were performed for 10 million generations, sampling every 1000 generations. The first 25% of sampled trees were discarded as burn-in. Convergence was assessed by ensuring an average standard deviation of split frequencies below 0.01 and effective sample sizes (ESS) greater than 200^25^. Posterior probabilities (PP) ≥ 0.95 were considered strong support^24^. Tree topologies obtained from MP and BI analyses were compared to evaluate clade stability.

**Table 1:** GenBank accession numbers of *Ganoderma* species sequences used in the phylogenetic analysis.

Species name	ITS rDNA GenBank accession No	Voucher/strain	Origin
*Ganoderma weberianum*	MZ354930	Dai19673	China
*Ganoderma weberianum*	MK603804	CBS21936	Philippines
*Ganoderma sichuanense*	MZ354928	Cui16343	China
*Ganoderma sichuanense*	MZ354929	Dai19651	Sri Lanka
*Ganoderma artocarpicola*	ON994239	HL173^T^	Yunnan, China
*Ganoderma artocarpicola*	ON994240	HL188	Yunnan, China
*Ganoderma bubalinomarginatum*	MZ354926	Dai20075^T^	Guangxi, China
*Ganoderma bubalinomarginatum*	MZ354927	Dai20074	Guangxi, China
*Ganoderma carocalcareum*	EU089970	DMC513	Cameroon
*Ganoderma carocalcareum*	EU089969	DMC322^T^	Cameroon
*Ganoderma parvulum*	MK554770	MUCL52655	Guiana, French
*Ganoderma parvulum*	MK554783	MUCL47096	Cuba
*Ganoderma austroafricanum*	MH571693	CMW25884	South Africa
*Ganoderma austroafricanum*	KM507324	CBS138724^T^	South Africa
*Ganoderma hoehnelianum*	MG279178	Cui13982	Guangxi, China
*Ganoderma hoehnelianum*	KU219988	Dai11995	Guangxi, China
*Ganoderma polychromum*	MG654197	MS330OR	OR, USA
*Ganoderma polychromum*	MG654196	MS343OR	OR, USA
*Ganoderma platense*	AH008110	BAFC2374	Spain
*Ganoderma platense*	AH008109	BAFC384	Spain
*Ganoderma sessile*	MG654307	113FL	USA
*Ganoderma sessile*	MG654306	111TX	USA
*Ganoderma suae*	PP869243	L4651^T^	Yunnan, China
*Ganoderma suae*	PP869244	L4817	Yunnan, China
***Ganoderma suae***	**PV335935**	**PU1013**	**Cao Bang, Vietnam**
*Ganoderma resinaceum*	MK554786	MUCL52253	France
*Ganoderma resinaceum*	MG706249	LGAM448	Greece
*Ganoderma resinaceum*	MG706250	LGAM462	Greece
*Ganoderma resinaceum*	MK554772	MUCL38956	Greece
*Ganoderma philippii*	MG279188	Cui14443	China
*Ganoderma philippii*	MN401410	MFLU19	Thailand
*Ganoderma tropicum*	ON994253	HL186	Yunna, China
*Ganoderma tropicum*	MG279194	Dai16434	Hainan, China
*Ganoderma phyllanthicola*	PP869246	HL308	Yunna, China
*Ganoderma phyllanthicola*	PP869245	L4948^T^	Yunna, China
*Amauroderma rugosum*	KJ531664	Cui9011	Guangdong, China

Phylogenetic relationships of *Pleurotus tuber-regium* were inferred from ITS rDNA sequences using the Maximum Likelihood method under the Tamura–Nei model^27^ in MEGA v12^26^. Branch support was assessed with 100 bootstrap replicates (values ≥ 60% shown). Rate heterogeneity among sites was modeled with a discrete gamma distribution (+ G, α = 0.6493) and a proportion of invariant sites (+ I = 27.56%). The dataset comprised 29 sequences and 1,397 aligned positions. The tree was rooted with *Hohenbuehelia mastrucatus*, and the scale bar represents substitutions per site (Table 2).

**Table 2:** GenBank accession numbers of *Pleurotus* species sequences used in the phylogenetic analysis.

Species name	ITS rDNA GenBank accession No	Voucher	Locality
*Pleurotus tuber-regium*	KP325386	PT120	Nigeria
*Pleurotus tuber-regium*	KP325382	N	Nigeria
***Pleurotus tuber-regium***	**PV335933**	**PU1010**	**Vietnam**
***Pleurotus tuber-regium***	**PV335934**	**PU1012**	**Vietnam**
***Pleurotus tuber-regium***	**PX067353**	**PU1011**	**Vietnam**
*P. abalonus (as P. cystidiosus var. abalonus)*	AY315806	CBS 803.91	China
*P. albidus*	KF280339	CCIBt 2405	Brazil
*P. albidus*	AF345661	TENN57623	Brazil
*P. australis*	AY315758	VT1953	Australia
*P. citrinopileatus*	JN234853	FSCC1 (PCY1)	Malaysia
*Pleurotus pulmonarius*	HM561984	FSC2	Malaysia
*P. cornucopiae*	AY265817	CBS 383.80	Netherlands
*P. cornucopiae*	DQ342325	PHZAU19	China
*P. cystidiosus*	AY315767	D420	USA
*P. djamor*	KF280328	CCIBt 2854	Brazil
*P. djamor*	GU722266	ECS-0128	Mexico
*P. dryinus*	EU424293	CBS 724.83	Netherlands
*P. eryngii*	U04089	D625	Unknown
*P. fuscosquamulosus*	KF280331	CCIBt 2404	Brazil
*P. ostreatus*	EU424300	CBS 593.82	Unknown
*P. pulmonarius*	KF280340	CCIBt 2963	Brazil
*P. pulmonarius*	AM269807	JPL-531-Sp	USA
*P. populinus*	AY368667	ATCC 90,083; KCCM 60,292	USA
*P. populinus*	U04095	D765	USA
*P. rickii*	KF280341	SP445788	Brazil
*P. smithii*	AY315779	IE74	Mexico
*P. smithii*	AY315780	CBS 680.82	Mexico
*Hohenbuehelia mastrucata*	EF409736	T-025	Canada

## Results

### Taxonomy

#### ***Ganoderma suae ***He et al.^6^

*Private number* PU1013.VIETNAM. *Collection location* Núi thủng Đoài Dương, Trùng Khánh District, Cao Bằng. *Date of collection* 14/05/2023, 11:32 CH GMT + 07:00. *Persons collected* L.T.H. Yen, Luane P, T. B. Tram and P.X.B. Minh. *Environment parameters* Temperature of 20 °C and 82% of humidity. *Habitat* Solitary, on a branch on the ground. *Odor* and taste Medicinal mushroom favor.

**Fig. 1:**
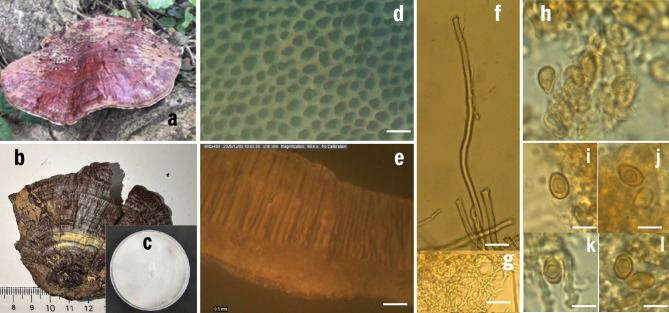
Morphological characteristics of *Ganoderma suae* PU1013: (**a,b**). Basidiomata in nature and dried; (**c**). Colony morphology after 7 days, (**d**). Hymenophore of the basidiocarp, (**e**). Longitudinal section of the pileus; (**f**). Skeletal hyphae and binding hyphae from context (magnification 40 ×); (**g**). Generative hyphae from tubes; (**h**). basidioles and basidiospores; (**i–l**). basidia. (**a–c**): taken by Iphone 12; (**d–e**): taken by Olympus SXZ12; (**f–g**): taken under Carl Zeiss AXIO Plant II (40 ×); (**h–l**): taken under Carl Zeiss AXIO Plant II (100X); Scale bar: (**d–e**): 0.5 mm; (**f–l**): 5 µm.

*Morphological description* Basidiomata annual, sessile, shell-shaped, applanate, becoming hard corky to woody hard upon drying. Pileus (Fig. 1a) broadly flabelliforme to semicircular, surface reddish brown with violet brown hues, strongly laccate, glabrous, displaying conspicuous concentric zones and slightly radial furrows, margin obtuse to slightly wavy. Pileus size up to approximately 5–9 cm in width, 4–7 cm in length, and 5–8 mm in thickness (Fig. 1a).

*Pore surface* cream-white when fresh, becoming pale yellowish to light brown when dry, slightly darkening upon bruising (Fig. 1b). Pores small, circular to slightly angular, 4–6 pores per mm (Fig. 1c). Tubes stratified, up to 8 mm long, homogenous context without black melanoid lines, context color light brown to yellowish brown, fibrous and corky when dry (Fig. 1b). Context greyish brown, homogeneous, without black melanoid lines, hard corky, up to 2 mm thick.

Tubes pale brown to pale grey, non-stratified, up to 6 mm long. Hyphal system trimitic; generative hyphae with clamp connections; all hyphae IKI −, CB + ; tissues darkening in KOH. Generative hyphae in context colourless, thin-walled, 2–3.5 μm diam; skeletal hyphae in context pale yellowish brown, thick-walled with a wide to narrow lumen or sub-solid, frequently arboriform and flexuous, 2.5–6 μm diam (Fig. 1e); binding hyphae in context colourless, thick-walled, branched and flexuous, 2–3 μm diam. Generative hyphae in tubes colourless, thin-walled, slightly swollen at the distal end, 2–3.5 μm diam; skeletal hyphae in tubes pale brown to dark brown, thick-walled, 2–6 μm diam. Cystidia and cystidioles absent.

Basidia clavate, ventricose, colourless, thin-walled, 15–35 × 13–18 μm, bearing 4 sterigmata; basidioles in shape like the basidia, colourless, thin walled, 14–25 × 9–15 μm. Basidiospores are broadly ellipsoid with thick, ornamented (verrucose) walls, brown in color, with a hyaline germ pore, dimensions: 5–7 × 4–5 µm (Fig. 1d).

The colony is white, with a smooth and velvety texture, growing relatively fast on PDA (Potato Dextrose Agar), reaching a diameter of 55–60 mm after 7 days of incubation at room temperature. The surface of the colony is uniform with an entire margin. In the aging stage, the colony undergoes lignification, developing a characteristic yellowish-brown coloration (Fig. 1f).

*Materials examined.* Vietnam, Cao Bang, Nui Thung, on wooden material on the ground, 14/05/2023, PU1013—Biological Resources Center (BRC)—Pheinkaa University.

*Suggested identification.* Polyporales*,* Polyporaceae*, Ganoderma suae.*

#### Pleurotus tuber-regium (Lentinus tuber-regium) (Fr.) Singer 1951

**Fig. 2:**
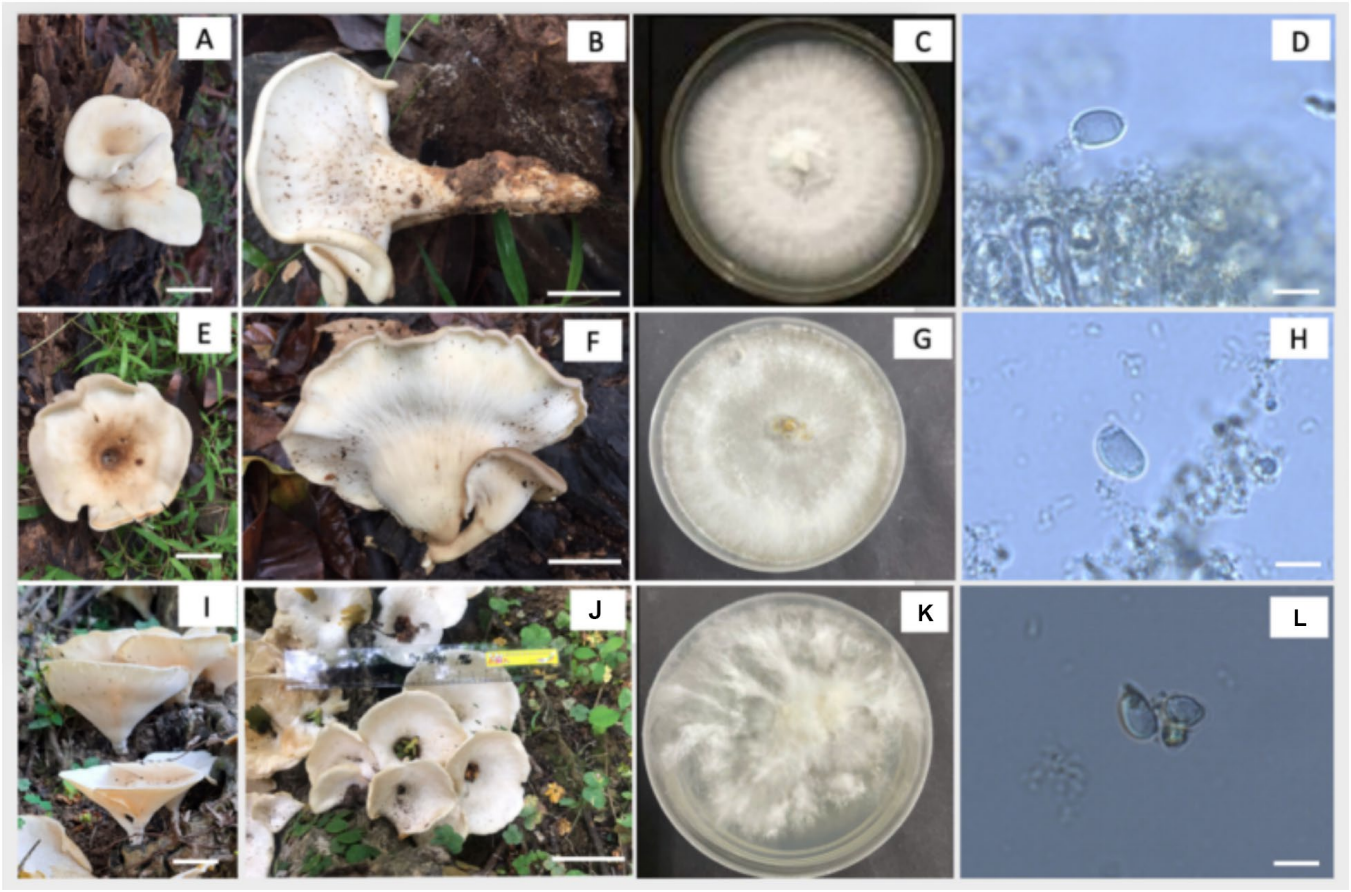
Morphological characteristics of *Pleurotus tuber-regium* (*Lentinus tuber-regium*). (**A–D**). *Pleurotus tuber-regium* PU1010 isolated in Da Phuc Temple, Nha Trang Khanh Hoa in July 2022. (**E–H**). *Pleurotus tuber-regium* PU1011 isolated Hon Ba, Nha Trang, Khanh Hoa in Oct 2024 and (**I–L**). *Pleurotus tuber-regium* PU1012 isolated in Nui Thung, Trung Khanh, Cao Bang in July 2023. (**A, B, E, F, I, J**). Fruiting body of *Pleurotus tuber-regium,* (**C, G, L**) colony of *Pleurotus tuber-regium* on PDA medium after 6 days culture. (**D, H, L**). Basidiospore of *Pleurotus tuber-regium.* (**A, B, E, F, J**) and (**K**) bar = 2.5 cm; (**D, H, L**) bar = 5 µm.

Three *Pleurotus tuber-regium* have been discovered in Nha Trang, Khanh Hoa, Vietnam. *Private number* PU1010 and PU1011 *Collection Location*: Tam Phuc Temple, Nha Trang, Khanh Hoa. Date of collection. July 2022 and Oct. 2024, respectively. *Persons collected*. L. T. H. Yen and K. T. An. Temperature of 26.5–27 °C and 84–86% of humidity, after rain. *Habitat*. Grow at the bottom of a decaying tree (Fig. 2A, B, E, F). *Odor and taste*. Distinctive, characteristic of edible mushrooms.

*Private number* PU1012, *Collection location* Núi thủng Đoài Dương, Trùng Khánh District, Cao Bằng. *Date of collection*. 14/05/2024. *Persons collected*. L. T. H. Yen, Luane P, T. B. Tram and P. X. B. Minh. *Environment parameters*. Temperature of 20 °C and 82% of humidity. *Habitat*. not solitary, Grow on decomposed wood buried underground. *Odor and taste*. Not distinctive (Fig. 2I,K).

*Morphological description*. Three above collected mushrooms have similar morphology as follows: The fruiting body of the mushroom has a funnel shape with wavy or flat edges, ivory to light brown in color, and the underside is white, consisting of densely packed, large, thin gills. The diameter of the pileus ranges from 7 to 9 cm under natural conditions, and reduces to 5–7.5 cm upon drying. The hymenophore is composed of crowded, decurrent lamellae, white to creamy in fresh specimens, becoming pale yellowish upon drying. Lamellae are thick, deeply descending along the stipe. The stipe is cylindrical and centrally positioned, measuring 10–20 × 1.5–2.0 cm, smooth, concolorous with the pileus, and sometimes tapering at the base. Basidiospores are ellipsoidal to ovate, smooth to finely rough, apically truncated, hyaline, thin-walled with an apically truncate apex, measuring 8–10.5 × 4.0–5.5 µm (Fig. 2D,H,M). Basidia are clavate**,** thin-walled, typically bearing four sterigmata, and measure 15–18 μm in length. The hyphal system is monomitic, comprising thin-walled, generative hyphae with abundant clamp connections.

*Colony characteristics*. All *Pleurotus* species exhibit a white colony color and show average growth on PDA medium. Colonies initiate with a dense, cottony center**,** progressing towards even or slightly irregular margins. Texture varies from velvety to cottony**,** depending on strain origin. The *Pleurotus* PU1010 strain grows more uniformly and has a velvety texture compared to the others (Fig. 2C). The *Pleurotus* PU1011 strain exhibits irregular margin, with the with sparse centers and denser peripheries (Fig. 2G). The colony of *Pleurotus* PU1012 grows rapidly but lacks uniform, mixed-texture colonies, with thin, sometimes transparent centers and undulating margin (Fig. 2L).

### Phylogeny

#### *Ganoderma suae* He et al.^6^

To determine the phylogenetic placement of *Ganoderma* PU1013, analyses based on ITS rDNA sequences were conducted using Maximum Likelihood (ML) and Bayesian Inference (BI) methods. *Amauroderma rugosum*, a member of Ganodermataceae representing a genus phylogenetically distinct from *Ganoderma*, was selected as the outgroup to provide appropriate rooting of the tree.

**Fig. 3:**
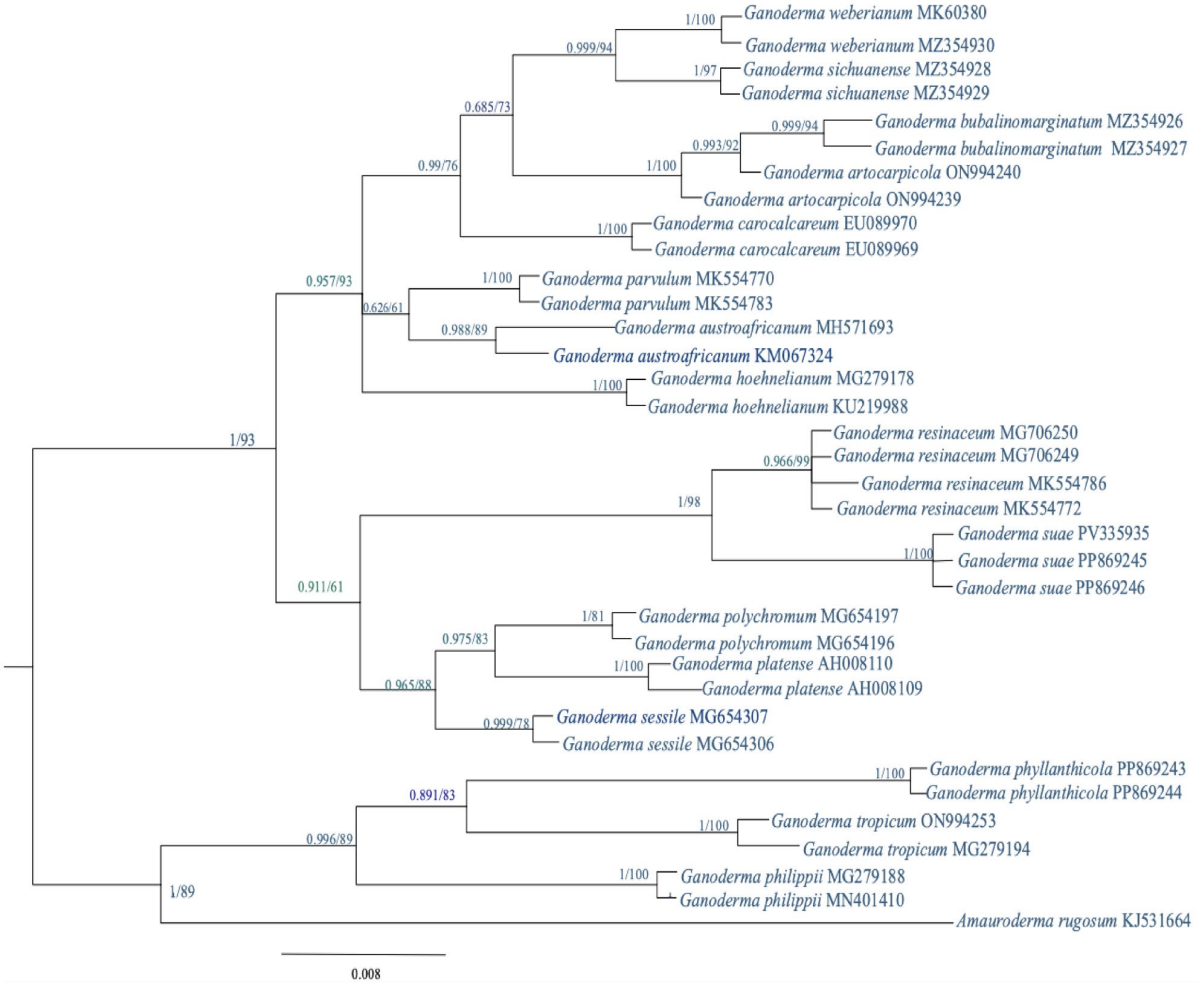
Maximum Parsimony (MP) and Bayesian Inference (BI) tree of *Ganoderma suae* PU1013 and related taxa inferred from ITS rDNA sequences. The tree was reconstructed using the Maximum Likelihood (ML) method under the Tamura–Nei^27^ substitution model. Node support values represent bootstrap percentages (BS) based on 100 replicates, and only values ≥ 60% are shown. *Amauroderma rugosum* was used as the outgroup. The scale bar indicates the number of nucleotide substitutions per site. Sequence accession numbers are shown next to the taxon names.

The results showed that strain PU1013 clustered within the *G. suae* clade^6^ with strong statistical support (ML bootstrap = 100%; BI posterior probability = 1; Fig. 3).

### Pleurotus tuber-regium (Fr.) Singer 1951

*Pleurotus* strains PU1010, PU1011, and PU1012 were identified based on phylogenetic tree construction using the Maximum Likelihood (ML) method. The results revealed that there was a highly supported monophyletic clade comprising the Vietnamese isolates with two reference sequences of *Pleurotus tuber-regium* (KP325386 and KP325382). This clade is supported by a bootstrap value of 100%, indicating robust phylogenetic confidence. Notably, the branch lengths between PU112, PU110, PU1011, and the reference strains are 0.00, suggesting no detectable genetic distance in the ITS region analyzed. This implies that these Vietnamese isolates are genetically indistinguishable from the reference sequences of *P. tuber-regium*, at least based on the ITS barcode. Such identical or near-identical sequences strongly support their placement within the *P. tuber-regium* clade. In contrast, other *Pleurotus* species such as *P. djamor* and *P. citrinopileatus* form distinct clades with greater branch lengths and lower similarity values, indicating clear genetic divergence (Fig. 4).

**Fig.. 4:**
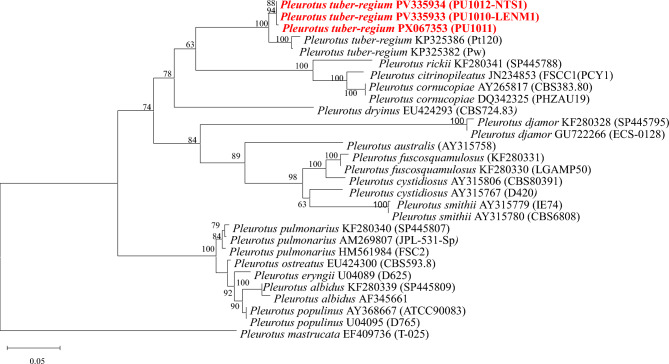
Maximum Likelihood (ML) phylogenetic tree of *Pleurotus tuber-regium* PU1010, PU1011 and PU1012 and related taxa inferred from ITS rDNA sequences. Bootstrap values > 60% are displayed on the corresponding branches. The scale bar is provided to indicate evolutionary distances. Sequences downloaded from GenBank are shown with their accession numbers.

## Discussion

### Ganoderma suae

The genus *Ganoderma* exhibits remarkable taxonomic and ecological diversity in Vietnam, reflecting the country’s rich fungal biodiversity and varied forest ecosystems. There were 14 species have been described: *Ganoderma colossus (Tomophagus colossus* (TC-02, Vietnam) is a white-rot species associated with large basidiocarps and tropical hardwood decay^10^; *Ganoderma weberianum* was collected on a *Cynometra dongnaiensis* tree in Ho Chi Minh City (BJFC029658–59) as reported in Sun et al^3^; *G. multipileum* isolated from dying *Delonix regia* metropolitan woody plant^3,13^, *Ganoderma cattienensis (Tomophagus cattienensis*), as reported by Le XT et al^10^ and Sun et al^13^ is a white-rot species associated with tropical hardwood decay and large basidiocarps; *Ganoderma applanatum* was identified by Cong^9^ as a white-mottled rot species with perennial fruiting bodies. *Ganoderma flexipes,* newly recorded from decaying hardwood roots, broadens the known ecological range of the species in tropical environments^27^. Other species such as *G. lucidum, G. lingzhi, G. luteomarginatum, G. subresinosum, G. phillipin, G. luteomarginatum, G. nasalanense* and *G. tropicum* were reported by Parmasto^7^, Kiet^8^, Nguyen and Khanh^11^; Viet Hung et al.^12^, Nguyen et al.^28^; Van On et al.^29^.

The above information confirms that, *Ganoderma* in Vietnam collectively occupy a broad ecological spectrum—ranging from primary evergreen forests and mountainous zones to urban shade trees—illustrating robust ecological plasticity and niche partitioning. The balance between temperate-affiliated lineages (e.g., *G. applanatum*) and tropically adapted clades (e.g., *G. colossus, G. cattienensis, G. multipileum*) reinforces Vietnam’s transitional biogeographic position, where northern subtropical and southern tropical microbiotas intersect.

Against this backdrop, the discovery of *Ganoderma suae* represents a significant expansion of the known ecological and geographic boundaries of the species. Previously documented only from Yunnan, China^6^, *G. suae* was not listed among Vietnamese species despite long-standing ethnomycological exploitation of *Ganoderma* mushrooms for medicinal use. The Vietnamese specimen PU1013 demonstrates clear conspecific affinity to the Chinese type based on combined macromorphological, micromorphological, cultural, and molecular characteristics.

*Macromorphological.* Both specimens produce annual, sessile basidiomata that are flabelliform to reniform, becoming corky to woody upon drying. The pileus surface in both collections is reddish brown to violet-brown, laccate, and concentrically zonate. The Vietnamese material exhibits pilei 5–9 cm wide and 4–7 cm long, slightly smaller than the Chinese type (up to 15 × 10 cm), but within natural intraspecific variation due to substrate and age differences. The context in both specimens is hard corky and fibrous, devoid of black melanoid lines, and shows a similar color range from light to cinnamon brown. Likewise, both have small circular to angular pores (4–6 per mm) and unstratified tubes measuring less than 1 cm, which are diagnostic of *G. suae*. The Vietnamese specimen lacks a stipe, while the type sometimes bears a short lateral or substipitate base up to 4.5 cm long. The absence of a stipe in PU1013 is considered ecologically variable, as substipitate forms of *Ganoderma* are frequently influenced by growth substrate or position on the host.

*Micromorphology.* The trimitic hyphal system of PU1013 with the generative hyphae is 2–3.5 µm diam., skeletal hyphae 2.5–6 µm, binding hyphae 2–2.5 µm, all IKI– and CB + /– matches precisely the diagnostic reactions and structural proportions of *G. suae*. The predominance of thick-walled, golden-yellow skeletal hyphae and the darkening of tissues in KOH were consistent in both. Basidia and basidioles in PU1013 are clavate to ventricose (15–35 × 13–18 µm) and (14–25 × 9–15 µm), somewhat similar in form to the type description (9–18 × 9–12 µm). The basidiospores of PU1013 (5–7 × 4–5 µm) are smaller than those of the Chinese type (9.0–10.5 × 5.5–6.5 µm). This reduction may reflect either developmental stage or local adaptation, as spore size is known to vary geographically within the *G. resinaceum* complex^2,3^. Nonetheless, both share identical features: double-walled, thick, verrucose (coarse-echinulate) endospore, CB + , IKI–, and possessing a hyaline germ pore—an unequivocal marker of *G. suae*^6^.

*Cultural characteristics.* The growth pattern of strain PU1013 on PDA—white, smooth, velvety mycelium that turns yellowish brown with age—corresponds closely to the color transition described by He et al.^6^ (“white to yellowish white, turning pale orange to reddish yellow after 3 weeks”). The colony diameter after 7 days (55–60 mm) and uniform margin further support its identity.

Molecular phylogenetic analysis based on the rDNA ITS region placed PU1013 firmly within the *Ganoderma suae* clade with maximal bootstrap support (100%; Fig. 3), confirming its close affinity with the ex-type strain of *G. suae*^6^. Within the phylogenetic framework of the *Ganoderma resinaceum* species complex as defined by Fryssouli et al^22^, PU1013 clustered within the well-supported *G. suae* lineage, although it exhibited a close relationship to G. resinaceum. Such proximity is consistent with the limited ITS divergence commonly observed among closely related taxa within this species complex. Although multilocus data were not available in the present study, the strong ITS-based clustering, together with congruent morphological characteristics, provides convincing evidence for assigning the Vietnamese specimen to *G. suae* rather than *G. resinaceum*. Nevertheless, expanded multilocus datasets in future investigations will be essential to further refine its phylogenetic placement and to assess potential intraspecific diversity within this recently described taxon.

This discovery represents the first record of *G. suae* in Vietnam, extending its known geographic range from Yunnan, China, into northern Indochina. The occurrence of *G. suae* in northern Vietnam highlights the continuity of subtropical hardwood fungal communities across the eastern Indochina—Yunnan floristic corridor. Like its Chinese counterpart, the Vietnamese specimen was collected on decaying hardwood-further supporting the ecological association of *G. suae* with broadleaf angiosperm hosts. The finding also resolves a previous misidentification of the specimen as *G. resinaceum*, reflecting the long-standing taxonomic complexity of the *G. resinaceum* species complex. Given the close phylogenetic relationship between *G. suae* and the well-studied medicinal mushroom *G. resinaceum*, similar bioactive properties and pharmacological potentials are anticipated. This result enriches the documented diversity of *Ganoderma* in Vietnam and provides a promising basis for future studies on its chemical constituents, cultivation, and medicinal applications.

### Pleurotus tuber-regium

*Known distribution and economic importance. Pleurotus tuber-regium* has been erected by Singer 1951 is an edible gilled fungus which is found on dead wood or under soil. It produces a sclerotium, or storage tuber, either within the decaying wood or in the underlying soil^30^. Their emerged sclerotia are round, dark brown with white interiors, and up to 30 cm wide. Both the sclerotium and the fruiting bodies are edible. It has a history of economic importance in Africa as food and as a medicinal mushroom^31^. Industrial cultivation is not yet common, but studies have shown *P. tuber-regium* can be grown on organic wastes such as corn, sawdust, cardboard, straw^31,32^,…. Mycelial growth occurs between 15 °C and 40 °C, with an optimum growth rate at 35°C^30^. The species somewhat wildely distributes in covering a diverse and very wide geographic range: from Africa (Chad, Kenya, Nigeria, Zambia, Uganda…) to Asia (India, Malaysia, Sri Lanka…) and into Oceania (Papua New Guinea, Solomon Islands, Australia)^31–34^. It is a widely recognized edible and medicinal mushroom species known for its bioactive potential and sclerotium-forming capability. It has been predominantly reported in tropical and subtropical regions, with confirmed distributions across Africa (e.g., Nigeria, Uganda, Zambia), Asia (e.g., India, Malaysia, Sri Lanka), and Oceania (e.g., Papua New Guinea, Solomon Islands, Australia)^31–35^.

*First confirmed record in Vietnam.* Laos, Thailand and Vietnam are situated in tropical zones which can be favorable for *Pleurotus* growth. Several pleurotoid mushrooms had been reported in Laos and Thailand, such as *Pleurotus djamor*, *Pl. eryngii*, *Pl. giganteus*, *Pl. ostreatus*, *Pl.* aff. *ferulaginis*, and *Pl. pulmonarius* in Laos, and *Pl. giganteus* and *Pl. sirindhorniae* in Thailand. Additional species of the genus *Lentinus* (e.g., *L. sajor-caju*, *L. squarrosulus, L. arcularius, L. polychrous*) were also documented, but not *P. tuber-regium*. But there were no reports that confirmed the occurrence of *P. tuber-regium* in Laos and Thai Lan^36,37^. In Vietnam, several *Pleurotus* had been reported, such as *P. pulmonarius*, *P. ostreatus*, *P. citrinopileatus, P. cystidiosus,* and* P. pulmonarius*^38^. However, until now, there were no reports that confirmed the occurrence of *P. tuber-regium* in Vietnam. In this study, we report for the first time the presence of *P. tuber-regium* in Vietnam, with three strains isolated from two distinct ecological regions: the Central region (Nha Trang) and the Northern mountainous area. The Central region, with a typical tropical climate, aligns well with the known ecological preferences of *P. tuber-regium* previously reported in Nigeria, Sri Lanka, and Malaysia. Remarkably, the detection of *P. tuber-regium* in the Northern highlands, where winter temperatures can drop to 8–15 °C, is of particular interest. Which could reflect the species’ adaptability to cooler conditions, similar to environments in parts of Australia. These findings not only expand the known geographical distribution of *P. tuber-regium* but also raise interesting questions about the species’ ecological flexibility and undocumented diversity in Southeast Asia. Furthermore, it is highly necessary to study the intraspecific divergence within *P. tuber-regium* species.

*Phylogenetic placement, genetic divergence, and morphology*. In this study, the three Vietnamese strains (PU1010, PU1011, PU1012) clustered together with other two known *P. tuber-regium* with bootstraps value of 100%. The ITS-based genetic divergence among Vietnamese strains was low, with pairwise similarity ranging from 99.80 to 100% (p-distance 0 – 0.00198) which falls within the expected range of intraspecific ITS variation reported for the species^37^. Morphological comparisons revealed no diagnostic differences among the Vietnamese collections, and minor variation in basidiome size or colony morphology is most plausibly attributable to environmental conditions rather than taxonomic differentiation.

*Bioactive potential and future research. Pleurotus tuber-regium* is widely recognized for its nutritional and pharmacological value. Both its sclerotia and fruiting bodies contain diverse bioactive compounds, including polysaccharides, phenolic constituents, triterpenoids, and mineral-rich matrices. Previous studies have demonstrated antioxidant, antitumor, antihypercholesterolemic, antihypertensive, antimicrobial, hepatoprotective, anti-obesity, and prebiotic activities, highlighting its potential as a functional food and nutraceutical resource^36–40^.

The confirmation of *P. tuber-regium* in Vietnam provides new genetic and biological material for chemical and pharmacological investigation. Future research should focus on comparative profiling of bioactive metabolites in Vietnamese strains, evaluation of strain-specific biological activities, and optimization of cultivation conditions to enhance compound yield. Integrative approaches combining metabolomic analysis, genomic characterization, and functional bioassays will be necessary to determine whether regional populations exhibit differences in bioactive composition or therapeutic potential. Such studies may contribute to the sustainable development of locally sourced medicinal mushrooms and support the valorization of Vietnam’s fungal biodiversity.

## Conclusion

This study reports *Ganoderma suae* and *Pleurotus tuber-regium* for the first time in Vietnam, expanding the country’s fungal biodiversity records. *Pleurotus tuber-regium* is widely recognized for its nutritional and medicinal importance, being traditionally consumed as both a food and a functional ingredient with immunomodulatory, antioxidant, and cholesterol-lowering properties. In contrast, *G. suae*—a recently described member of the *Ganoderma resinaceum* complex—has not yet been investigated for its bioactive constituents. The confirmation of *G. suae* in Vietnam therefore provides new material for future studies on its chemical composition, cultivation potential, and possible medicinal applications. Together, these findings enrich the understanding of Vietnam’s macrofungal diversity and highlight the country’s potential as a reservoir of both established and emerging medicinal mushrooms. These findings open new opportunities for research, sustainable utilization, and commercialization. Further studies should explore their pharmacological applications and optimize cultivation techniques.

## Data Availability

The datasets generated and analyzed during the current study are available in the NCBI repository. Sequence data that support the findings of this study have been deposited in the NCBI with the accession codes: Sequence data that support the findings of this study have been deposited in the NCBI with the accession codes: SUB15183694: PU1010 PV335933 SUB15183694: PU1012 PV335934 SUB15183694: PU1013 PV335935 SUB15502447: PU1011 PX067353 Link to access the data as follow: https://www.ncbi.nlm.nih.gov/nuccore/PV335933 https://www.ncbi.nlm.nih.gov/nuccore/PV335934 https://www.ncbi.nlm.nih.gov/nuccore/PV335935 https://www.ncbi.nlm.nih.gov/nuccore/PX067353.
